# 555. Utilization of Remdesivir for COVID-19 in the National Veterans Affairs Healthcare System

**DOI:** 10.1093/ofid/ofab466.753

**Published:** 2021-12-04

**Authors:** Aisling Caffrey, J Xin Liao, Vrishali Lopes

**Affiliations:** 1 Rhode Island Infectious Diseases Research Program, Providence, RI; 2 Infectious Diseases Research Program, Providence Veterans Affairs Medical Center, Providence, RI; 3 College of Pharmacy, University of Rhode Island, Kingston, RI, Providence, RI; 4 PVAMC, Providence, RI

## Abstract

**Background:**

As remdesivir (GS-5734) has become a leading treatment for COVID-19, we sought to assess remdesivir utilization patterns, including utilization of concomitant and supportive therapies, and heterogeneity in treatment approaches.

**Methods:**

Our retrospective cohort study included hospitalized Veterans with positive COVID-19 PCR tests treated with remdesivir, from 03/2020 through 4/2021. Using exposure mapping of barcode medication administration records and medication dispensings, we assessed other medications received by each patient on each day of remdesivir treatment. Heterogeneity was defined as patterns of treatment (drug & duration) not shared by any other patient.

**Results:**

Our study included 13,665 patients with COVID-19 receiving remdesivir. The median time to remdesivir initiation from either positive test or hospital admission was 1 day (interquartile range [IQR] 0-4 and 0-1, respectively). The median duration of remdesivir treatment was 5 days (IQR 4-5 days). Median length of hospital stay was 7 days (IQR 4-13). Inpatient mortality was 13.9% and an additional 6.2% of patients died within 90 days of discharge. The most common concomitant and supportive therapies were anticoagulants/antiplatelets (94.8%; enoxaparin 72.6%, heparin 18.4%, apixaban 10.8%, clopidogrel 6.3%), corticosteroids (90.8%; dexamethasone 87.3%, prednisone 2.9%, methylprednisolone 5.5%), statins (55.8%; atorvastatin 38.2%, simvastatin 7.1%, rosuvastatin 6.0%), antibiotics (41.9%; azithromycin 25.6%, ceftriaxone 13.2%, doxycycline 6.0%, vancomycin 4.9%), angiotensin receptor blockers (11.9%) and angiotensin-converting enzyme inhibitors (20.4%), melatonin (29.7%), and aspirin (35.6%). Concomitant utilization of Janus kinase inhibitors (0.5%), interleukin-6 inhibitors (2.4%), and hydroxychloroquine (0.5%) was low. Heterogeneity in concomitant and supportive therapies during remdesivir treatment was 84.6% (68.3% when assessed as drug class/category).

**Conclusion:**

Among hospitalized patients with COVID-19 in the national VA Healthcare system receiving remdesivir, remdesivir was initiated early in the admission and substantial heterogeneity was observed in concomitant and supportive therapies during remdesivir treatment.

**Disclosures:**

**Aisling Caffrey, PhD**, **Merck** (Research Grant or Support)**Pfizer** (Research Grant or Support)**Shionogi, Inc** (Research Grant or Support)

